# T Cells' Immunological Synapses Induce Polarization of Brain Astrocytes *In Vivo* and *In Vitro*: A Novel Astrocyte Response Mechanism to Cellular Injury

**DOI:** 10.1371/journal.pone.0002977

**Published:** 2008-08-20

**Authors:** Carlos Barcia, Nicholas S. R. Sanderson, Robert J. Barrett, Kolja Wawrowsky, Kurt M. Kroeger, Mariana Puntel, Chunyan Liu, Maria G. Castro, Pedro R. Lowenstein

**Affiliations:** 1 Board of Governors' Gene Therapeutics Research Institute, Department of Medicine, Cedars-Sinai Medical Center, Los Angeles, California, United States of America; 2 Department of Medicine, The Brain Research Institute, Jonsson Comprehensive Cancer Center, David Geffen School of Medicine, University of California Los Angeles, Los Angeles, California, United States of America; 3 Department of Molecular and Medical Pharmacology, The Brain Research Institute, Jonsson Comprehensive Cancer Center, David Geffen School of Medicine, University of California Los Angeles, Los Angeles, California, United States of America; 4 Department of Endocrinology, Cedars-Sinai Medical Center, Los Angeles, California, United States of America; Karolinska Institutet, Sweden

## Abstract

**Background:**

Astrocytes usually respond to trauma, stroke, or neurodegeneration by undergoing cellular hypertrophy, yet, their response to a specific immune attack by T cells is poorly understood. Effector T cells establish specific contacts with target cells, known as immunological synapses, during clearance of virally infected cells from the brain. Immunological synapses mediate intercellular communication between T cells and target cells, both *in vitro* and *in vivo.* How target virally infected astrocytes respond to the formation of immunological synapses established by effector T cells is unknown.

**Findings:**

Herein we demonstrate that, as a consequence of T cell attack, infected astrocytes undergo dramatic morphological changes. From normally multipolar cells, they become unipolar, extending a major protrusion towards the immunological synapse formed by the effector T cells, and withdrawing most of their finer processes. Thus, target astrocytes become polarized towards the contacting T cells. The MTOC, the organizer of cell polarity, is localized to the base of the protrusion, and Golgi stacks are distributed throughout the protrusion, reaching distally towards the immunological synapse. Thus, rather than causing astrocyte hypertrophy, antiviral T cells cause a major structural reorganization of target virally infected astrocytes.

**Conclusions:**

Astrocyte polarization, as opposed to hypertrophy, in response to T cell attack may be due to T cells providing a very focused attack, and thus, astrocytes responding in a polarized manner. A similar polarization of Golgi stacks towards contacting T cells was also detected using an *in vitro* allogeneic model. Thus, different T cells are able to induce polarization of target astrocytes. Polarization of target astrocytes in response to immunological synapses may play an important role in regulating the outcome of the response of astrocytes to attacking effector T cells, whether during antiviral (e.g. infected during HIV, HTLV-1, HSV-1 or LCMV infection), anti-transplant, autoimmune, or anti-tumor immune responses *in vivo* and *in vitro*.

## Introduction

Astrocytes play an active role in maintaining the structure, metabolism, and function of the brain. They provide nutrients to neurons from endfeet located on brain endothelial cells, and participate actively in the blood-brain-barrier. They also ensheath neuronal synaptic junctions; here, astrocytes play an essential function in controlling the levels of the neurotransmitters glutamate and adenosine in the brain extracellular space, and thus, neuronal excitation[1]–[6].

Astrocytes are cells with multiple processes; their overall morphology has been classically described as ‘fibrous’ or ‘protoplasmic’, depending on their location within the white (fibrous) or grey matter (protoplasmic). In complex three dimensional reconstructions protoplasmic astrocytes appear as spongiform cells; their thicker multiple processes slowly becoming finer forming terminals surrounding either blood vessels or neuronal synaptic junctions[7]–[9].

In response to diverse brain injury, i.e. physical trauma, hypoxia, ischemia, stroke, or neurodegeneration, astrocytes become hypertrophic; such astrocytes are also referred to as reactive and/or activated astrocytes [7]–[9]. Reactive astrocytes are recognized through their increase in size, and upregulation of glial fibrillary acidic protein (GFAP) expression and immunoreactivity [5]. Influx of T cells into the brain and elimination of virally infected astrocytes could be considered another form of brain injury. Herein we demonstrate that virally infected astrocytes respond to a direct T cell attack in a novel manner [10]. Instead of a symmetrical hypertrophic size increase, infected astrocytes contacted by antiviral T cells respond by adopting a cellular morphology that is polarized towards the T cells. A similar polarized response was detected in non-infected primary astrocytes in culture upon exposure to allogeneic T cells. Thus, astrocyte polarization may be a generalized response to T cell attack, in cases of antiviral, anti-tumor, autoimmune, or anti-transplant immune responses.

Adenoviral vectors are powerful gene transfer tools for transgene expression in the brain. Adenoviral vectors transduce a variety of brain cells, including astrocytes, and allow long term widespread transgene expression if injected directly and carefully into the brain parenchyma [11]. If an immune response to adenovirus is pre-existing, or stimulated through a systemic administration of adenovirus, however, transgene expression and vector genomes are eliminated [10]–[12]. This immune response is one of the biggest challenges to gene therapy. In order to improve gen[13] therapy to the brain, we are interested in understanding the cellular and molecular mechanisms by which the immune system eliminates transgene expression from the brain.

Recently, we demonstrated that during the clearance of adenovirally transduced astrocytes from the brain, CD8^+^ T cells form close anatomical appositions with the target infected astrocytes [14], [15]. During this process, CD8^+^ T cells selectively invade the brain parenchyma where infected cells are located, and eventually eliminate approx. 50% of infected cells, 85% of which are GFAP+ astrocytes. Importantly, we determined that at the close anatomical appositions between CD8^+^ T cells and infected astrocytes [10], T cell membrane proteins such as the adhesion molecule lymphocyte function-associated antigen-1, LFA-1, and the T cell receptor, TCR, adopted *in vivo* patterns of distribution described previously *in vitro* as immunological synapses.

Immunological synapses are thought to organize and channel intercellular communication in the immune system [14], [16]–[21]. Immunological synapses form between naïve T cells and dendritic cells during T cell priming, and between CD8^+^ T cells and target cells during the effector stage of the immune response [16], [22]–[24]. In Kupfer-type synapses the TCR is localized to a central portion of the interface (i.e., central supramolecular activation cluster [c-SMAC]), while adhesion molecules such as LFA-1 form a ring, peripheral-SMAC (p-SMAC), surrounding the c-SMAC [10], [16], [19], [25], [26]. The cytoskeleton and secretory machinery of T cells also becomes polarized towards the immunological synapse; i.e. the microtubule organizer center (MTOC) and Golgi apparatus reorient towards the immunological synapse [26]–[30] in a process depending on cytoskeletal and motor proteins, such as dynein, tubulin and actin [16], [28], [29], [31]–[38]. The polarization of the T cell cytoskeleton and secretory machinery is thought to allow vectorial secretion of effector molecules at the immunological synapse, such that IFNγ and granzyme are secreted through the immunological synapse. Interestingly, other molecules, such as TNFα and RANTES are secreted outside the immunological synapse [17], [31], [39]. Most recently, we have also demonstrated that IFNγ becomes polarized at immunogical synapses formed *in vivo* between brain infiltrating CD8^+^ T cells and adenovirally infected astrocytes. This strongly suggests that immunological synapses *in vivo* are the anatomical conduit of intercellular communication between effector T cells and their targets[39].

In the present work, we demonstrate that target astrocytes became polarized in response to T cell attack (whether the T cells are anti-viral, or allogeneic T cells); as a result, astrocytes altered their morphology, shape, internal cytoskeletal and organelle organization, and distribution of selected membrane proteins. The target cells became unipolar, with one main process extending up to 20 μm in length, and directed towards the contacting T cell. Other cellular processes appeared to be retracted. Intracellular organelles became reoriented towards the T cell immunological synapse; the MTOC and Golgi from the target astrocyte reoriented towards the immunological synapse. These results demonstrate that T cells interacting with target virally infected astrocytes through immunological synapses induce major rearrangements in the organization of target cells in the brain; a similar polarization of intracellular organelles towards immunological synapses was also detected in an allogeneic T-astrocyte interaction *in vitro*. Of note, such rearrangements in response to T cell attack appear to differ significantly from hypertrophic changes in astrocyte morphology induced by trauma, hypoxia or neurodegeneration representing a novel mechanism of astrocyte response to injury. Thus, understanding the cellular and molecular basis of astrocyte responses to T cell attack, could lead to new insights into our understanding of the mechanisms of how the immune system clears viral infections, transplants, or tumors, from the brain, with potential implications for our understanding and treatment of brain infections including HIV/AIDS, HTLV-1, HSV-1, West Nile virus, as well as clinical implications for understanding the role of the immune system in neurological disorders such as brain cancer, autoimmune brain diseases, and transplantation for the treatment of Parkinson's disease.

## Materials and Methods

### Adenoviral vectors

Adenoviruses used in this study were first-generation E1/E3-deleted recombinant adenovirus vectors based on adenovirus type 5. The construction of Ad-TK (expressing herpes simplex virus type I thymidine kinase, HSV1-TK) and Ad-HPRT (expressing hypoxanthine-guanine phosphoribosyl-transferase), both transgenes under the major immediate early human cytomegalovirus promoter (hCMV), has been described in detail elsewhere [13], [40].

### Animals, surgical procedures, viruses

All experimental procedures were carried out in accordance with the NIH Guide for the Care and Use of Laboratory Animals and approved by Cedars-Sinai Medical Center Institutional Animal Care and Use Committee (CSMC IACUC). *In vivo* studies used adult male Sprague-Dawley rats (250g body weight) (Charles River). Animals were injected unilaterally in the left striatum with 1×10^7^ i.u. of Ad-TK in a volume of 1 μl at day 0 in order to infect astrocytes with adenoviral vector. One month later, rats were anaesthetized briefly and injected intradermally with 100 μl of sterile saline containing 5×10^8^ infectious units of Ad-HPRT. Animals were sacrificed 14 days after immunization, which is the time point of maximum infiltration of CD8^+^ T cells in the brain parenchyma and the maximum peak of interaction between virally infected cells and T cells [10]. Animals were anesthetized and transcardially perfused with 200–500 ml of oxygenated Tyrode's solution. Immediately afterwards, animals were perfused with 4% paraformaldehyde to fix the brain. Brains were sectioned on a vibratome (Leica Instruments, Exton, PA) at 50 μm section thickness. Primary cultures were generated from neonatal (postnatal day 4) pups obtained from timed pregnant Sprague-Dawley females (Charles River). Splenocytes were obtained from adult female Sprague-Dawley or adult male Lewis rats.

### Allogeneic astrocyte/splenocyte co-cultures

Cerebral cortices were dissected from outbred Sprague-Dawley pups, dissociated with trypsin and cultured in astrocyte medium [DMEM supplemented with 10% fetal bovine serum (FBS), 50 units/ml penicillin, 50 μg/ml streptomycin, 1mg/ml glucose] in poly D-Lysine-coated flasks for eight days, then seeded onto coverslips in 24-well plates of the same medium. Twenty four hours later, splenocytes obtained from spleens of adult rats (Lewis or Sprague-Dawley) after overdose of ketamine and xylazine were resuspended in RPMI-10 (RPMI, 10% FBS, antibiotics as above) and added to the wells at a ratio of 10:1 splenocytes to astrocytes. At time points ranging from 20 minutes to 24 hours subsequently, coverslips were transferred to PBS at 37°C, fixed in 4% paraformaldehyde at room temperature for 10 mins, washed twice in PBS and stored at 4°C in PBS, 0.1% sodium azide until immunofluorescent labeling and morphological analysis. Sprague-Dawley rats are an outbred strain; thus, the combination of either Lewis or Sprague-Dawley splenocytes with Sprague-Dawley astrocytes (wherein the splenocytes or astrocytes are always from different individuals), constituted allogeneic combinations. All media and tissue culture reagents were obtained from Cell-Gro (Manassas, VA) except for FBS which was obtained from Omega Scientific (Tarzana, CA).

### Immunocytochemical procedures and confocal analysis

50 μm coronal brain sections were cut serially through the striatum, and immunofluorescent detection was performed as described in detail earlier [10], using the following primary antibodies recognizing: CD8 (1∶500; mouse, Serotec), TK (1∶10,000; rabbit, custom made by our laboratory), TK (1∶1,000; Chicken, custom made by AvesLabs Inc.), NeuN (1∶1,000; mouse, Chemicon), GFAP (1∶500; guinea pig, Advanced Immunochemical), LFA-1 (1∶500; mouse, IgG2a, BD Pharmingen), TCR (1∶100; mouse, IgG_1_, BD Pharmingen), MHC-I (1∶1,000; mouse, IgG_1_, Serotec), GM130 (1∶1,000; mouse, IgG2a, Abcam), α-Tubulin (1∶500; mouse, Sigma), γ-Tubulin (1∶500; rabbit, IgG_1_, Sigma), glutamate transporter 1, GLT-1 (1∶500; Mouse, IgG1 , BD Biosciences), Connexin 43: (1∶50; Rabbit, Cell Signaling), Aquaporin 4: (1∶500; Rabbit, Chemicon), glutamate aspartate transporter, GLAST (1∶100; Rabbit, Abcam), mGluR5: (1∶200; Rabbit, Upstate), active caspase-3 (1∶1000; Rabbit, Promega).

Immunocytochemical detection methods were optimized during preliminary experiments to achieve full and homogenous antibody penetration throughout the total thickness of vibratome sections. Adjacent 50 μm thick sections of each brain were pretreated with citrate buffer for 30 min at 65°C to increase antigen retrieval and penetration of the antibodies into the tissues. Sections were permeabilized with 1% Triton X-100 for 5 min and blocked with 3% normal horse serum in 0.1 M PBS, pH 7.4, for 60 min. Sections were incubated at room temperature for 48h with primary antibodies combined followed by 4 h of incubation with the appropriate secondary antibodies, Alexa 488, Alexa 546, Alexa 594 and/or Alexa 647 (1∶1,000; Invitrogen). For F-actin staining sections were incubated with Alexa Fluor 488-phalloidin (1∶500 in PBS; Invitrogen) during two hours at room temperature after immunostaining.

After washing, sections were incubated with DAPI solution for 30 min. Sections were washed, mounted, and examined using both fluorescent and confocal microscopy. Brain sections were examined using a Leica DMIRE2 confocal microscope (Leica Microsystems, Exton, PA) with 63× oil objective and Leica Confocal Software (Leica Microsystems Heidelberg 19 GmbH). A series range for each section was set by determining an upper and lower threshold using the Z/Y Position for Spatial Image Series setting, and confocal microscope settings were established and maintained by Leica and local technicians for optimal resolution. Contacts were defined as areas where co-localization of both markers occurs between two cells in at least two 0.5 μm thick optical sections. Contacts can also be illustrated as they appear throughout the stack of sections as a simple 0.5 μm layer or as a transparency of all layers merged together. Three dimensional reconstructions to allow rotation of the images were rendered with alpha blending software (custom made by KW). More than 150 immunological synapses at various stages of development from 20 animals were recorded and analyzed in detail.

Immunocytochemical labeling of cell cultures on coverslips was essentially similar to that described for tissue sections, using the same antibodies, but with primary and secondary antibody incubation times reduced to 2–4 hours, and Triton-X concentration in buffers reduced to 0.02%. For *in vitro* staining actin was detected using Alexa-594-conjugated phalloidin (Invitrogen).

### Quantification of morphological changes in target virally infected astrocytes

**Figure 1:**
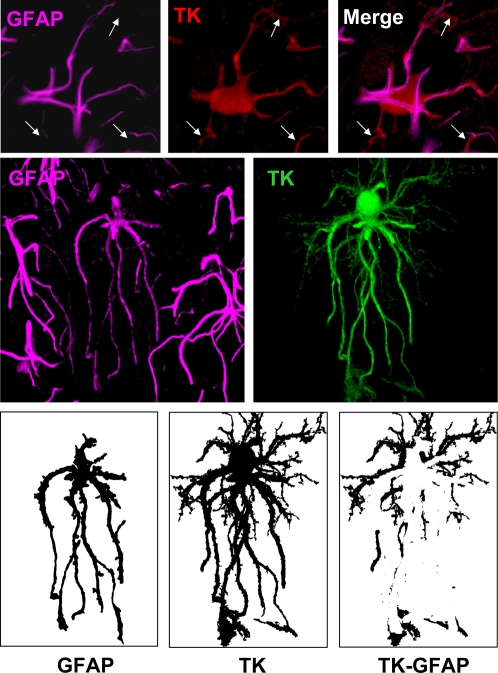
Immunocytochemistry for TK reveals the full extent of infected astrocyte morphology compared to GFAP. Confocal images show double staining of TK and GFAP in rat brain sections. The top row shows that TK and GFAP stain different structures and processes of the same cell. Arrows indicate processes that are TK+ but not GFAP+. The middle row shows a TK+ cell which is also GFAP+. Note that TK staining reveals more processes, and a fuller cell body than GFAP. In the lower row we show the optical segmentation of TK, GFAP and the non-overlapping area (GFAP image subtracted from TK image). The result shows the TK+ processes that are not GFAP+. This demonstrates that TK provides a fuller image of infected astrocytes.

The number of astrocyte TK-expressing processes and protrusions and their diameters were measured with Leica Confocal Software (Leica Microsystems, Heidelberg, Germany). We choose TK as the most reliable marker for cell body and processes since other markers, like GFAP, do not mark all cell processes making it difficult to interpret the cell morphology (Figure 1).

**Figure 2:**
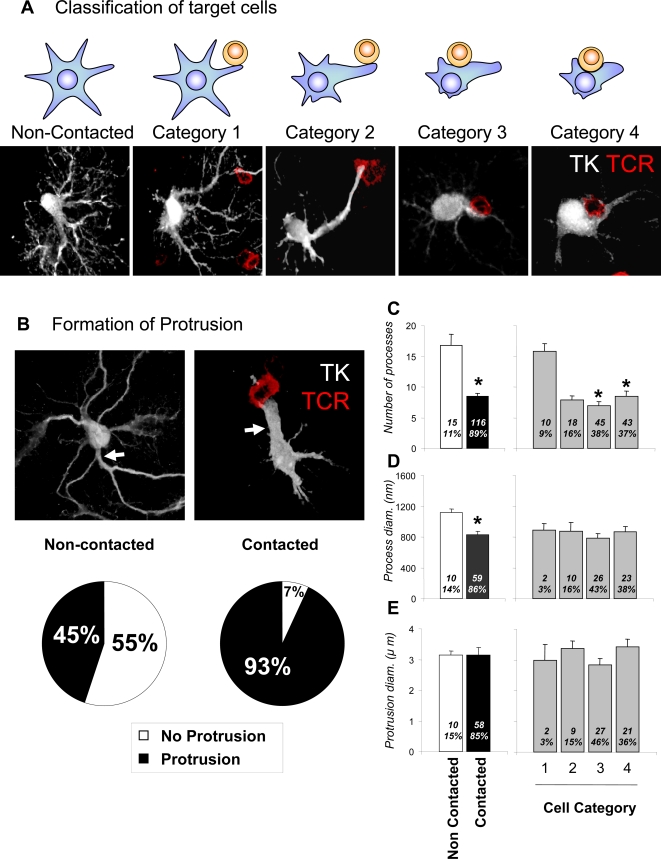
Virally infected astrocytes display a different phenotype when contacted by T cells. Astrocytes contacted by infiltrating T cells display various alterations in morphology in comparison with cells not contacted. According to the degree of morphological alteration, the morphologies of contacted cells were further classified into the four categories illustrated in (A). In (A) target astrocytes are represented as blue stellate shapes, while contacting T cells are represented as round yellow shapes. The confocal micrographs illustrate Category 1–4 infected target cells immunolabeled for TK (white) and T cells immunolabeled for TCR (red). Category 1 includes virally infected cells (i.e., expressing TK) with T cells contacting regular processes (<2 μm in diameter); Category 2 includes virally infected cells with T cells contacting a protrusion (a cell process >2.5 μm in diameter); Category 3 includes virally infected cells with T cells in contact with the cell body with obvious cytoplasm between the cells; and Category 4 includes virally infected cells with T cells in contact with the cell body without obvious cytoplasm between the two cells. (B) shows confocal images of virally infected cells (TK, white) either contacted by T cells (right panel, TCR, red), or non-contacted (left panel). A significantly higher proportion of virally infected, contacted cells displayed protrusions (93%) when compared to virally infected but non-contacted cells (45%) (p<0.05, Chi square test); white arrows indicate a branching protrusion in a non-contacted astrocyte in the left panel, and an astrocyte protrusion contacting a T cells in the right panel. (C) shows the number of processes, (D) the diameter of the processes, and (E) the diameters of protrusions in virally infected cells, either contacted by T cells or non-contacted. The left histograms in (C) through (E) compare contacted versus non-contacted cells; 1–4 are categories as described in (A). Numbers and percentages of cells in each category are indicated inside each bar. Note that the number of processes and their diameters are significantly reduced in contacted cells (* p<0.05; Student's t test). The reduction in process number is evident in categories 2 and higher (C, * p<0.05 v. Category 1; one way ANOVA), while the reduction in process diameter is seen in all categories of contacted cell.

The morphology of infected astrocytes was analyzed by the expression of TK; infected astrocytes in contact with T cells were classified in different categories according to their morphology and their relationship to T cells. The classification was made considering: i) the presence or absence of T cells in the vicinity of infected astrocytes; ii) the site at which T cells contact infected astrocytes; and iii) the presence or absence of a larger astrocyte extension, named a protrusion, and defined as a process >2.5 μm in diameter; all cellular extensions <2.0 μm diameter are referred to as processes. Cells were classified as follows: Category 1-virally infected cells (i.e., expressing TK) with T cells contacting regular processes (<2 μm in diameter); Category 2-virally infected cells with T cells contacting a protrusion (>2.5 μm in diameter); Category 3-virally infected cells with T cells in contact with the cell body with obvious cytoplasm between the cells; and Category 4-virally infected cells with T cells in contact with the cell body without obvious cytoplasm between the two cells (Figure 2A).

Relative fluorescence intensity along different structures and across the plane of the immunological synaptic interface was measured with Leica confocal software, and is illustrated in the figures with corresponding arrows traversing the measured optical planes.

Length of Golgi in virally infected cells was measured as the extent of the GM130-immnureactive mass from around the cell nucleus to the farthest point in the cytoplasm. The displacement of the MTOC in virally infected cells was similarly measured as the distance between the external border of the nucleus and the farthest point of γ-Tubulin expression. 160 immunological synapses were analyzed for Golgi and MTOC quantification.

Quantification of MHC-I was performed in astrocytes' regular processes and protrusions as well as across the surface of the entire cell. In processes and protrusions, three measurements were made: Two sides of the process (M1 and M2) and cytoplasm (C) were measured. 29 MHC-I positive cells were measured and more than 150 fluorescence measurements were made. Data were expressed as the mean ±SEM.

### Statistical analysis

The statistical significance of data was analyzed using a Chi square test, student's t test, or one-way ANOVA followed by Tukey post-test. When data failed normality test and Levene equal-variance test, they were analyzed by the non-parametric Kruskal-Wallis test followed by Dunn's post-test. Differences were considered statistically significant if p<0.05.

## Results

### Immunohistochemistry for TK reveals the full extent of infected astrocyte morphology, in a more complete manner than GFAP

Immunofluorescent detection of thymidine kinase (a cytoplasmic protein) expressed from the viral vector labeled the cytoplasm of infected astrocytes more completely than GFAP (which forms part of intermediate filaments). In general immunoreactivity for both proteins could be detected in the larger processes, but in many of the finer processes, TK immunoreactivity was visible while GFAP was not, and TK immunoreactivity generally occupied a greater area of the soma than did GFAP (Figure 1).

### Antiviral effector T cells establish close contacts with infected astrocytes and induce the formation of a protrusion and a reduction in the total number of processes

Astrocytes in contact with CTLs underwent complex morphological changes. There was a statistically significant reduction in the number of astrocyte processes when astrocytes were in contact with CTLs (Figure 2A, C), and a significant increase in the number of protrusions (astrocytic processes of diameter >2.5 μm; Figure 2A, B). Such protrusions were found in 93% of virally infected astrocytes contacted by T cells, (Figure 2B), a significant difference when compared to non-contacted cells, of which only 45% were found to possess protrusions. Importantly, processes of diameter >2.5 μm in non-contacted cells were short, and gave rise to smaller diameter processes; protrusions in contacted cells were longer (data not shown), and did not branch (see white arrows in Figure 2B); there was no significant difference in the diameter of protrusions between contacted and non-contacted cells. In asrocytes in direct contact with the CTL (Figure 2B) the protrusions were always oriented towards the T cells.

There was a statistically significant decrease in the diameter of astrocytes' processes that were not in contact with CTLs (Figure 2D). Non-contacted, virally infected astrocytes generally possessed a considerable number of ramified processes which were mostly of a similar diameter (Figure 2B).

### Antiviral effector T cells establish close contacts with infected astrocytes and induce the redistribution of astrocyte organelles towards the major protrusion and/or the attacking T cell

#### i Golgi Apparatus

**Figure 3:**
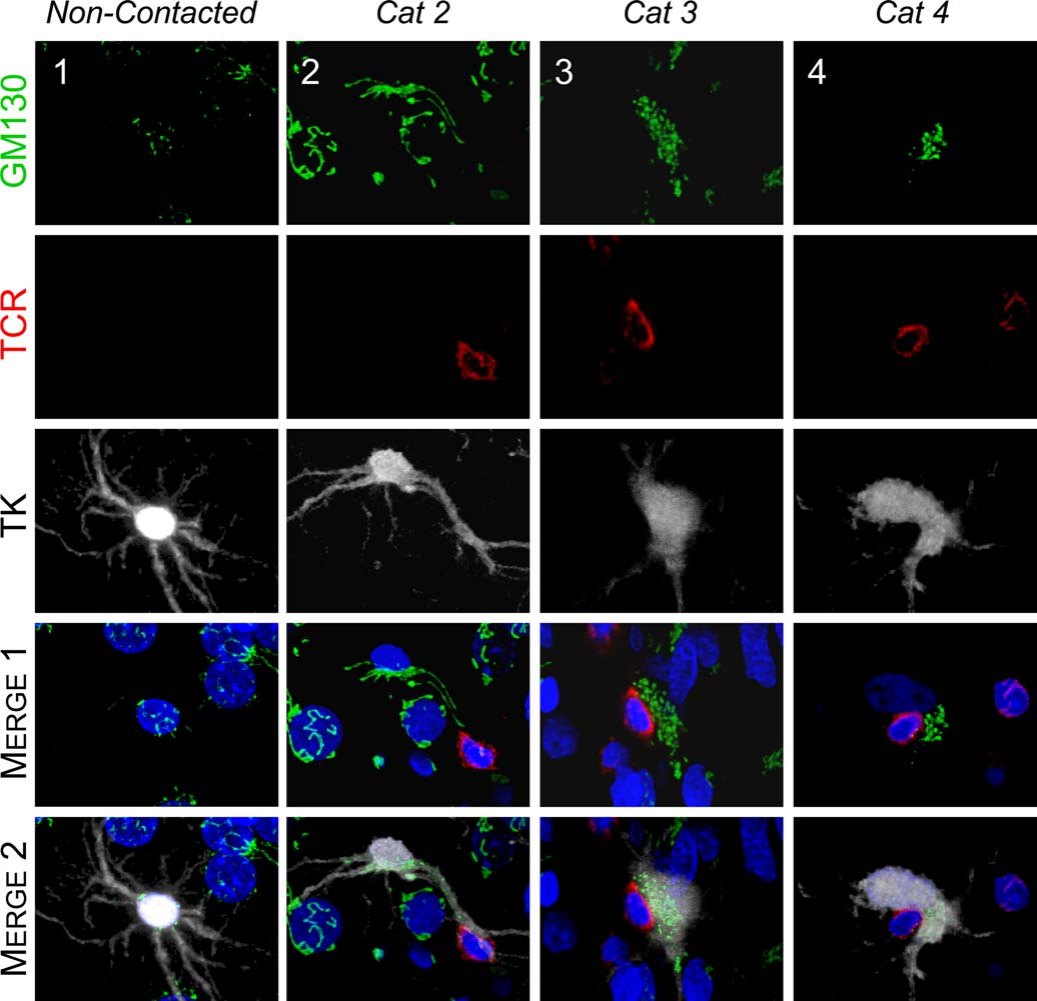
The Golgi apparatus of infected brain cells is polarized towards the T cell in immunized animals. Representative confocal images of infected brain cells and brain-infiltrating T cells are shown. Immunocytochemistry was performed with markers for cis-Golgi apparatus (green, GM130), T cells (red, TCR), virally infected brain cells (white, TK), and nuclei (blue, DAPI). MERGE 1 and MERGE 2 show the spatial relationship between the Golgi apparatus of virally infected cells and the contacting T cells. Note that the Golgi apparatus in non-contacted cells (Column 1) is perinuclear, and does not appear to enter astrocyte processes. The Golgi apparatus of Category 2 cells (Column 2) is hypertrophic, localized inside the protrusion, and polarized towards the T cell. Column 3 shows a Category 3 cell with hypertrophic Golgi apparatus polarized towards the T cell. The Golgi apparatus of a Category 4 cell (Column 4) is condensed to an area adjacent to the T cell.

**Figure 4:**
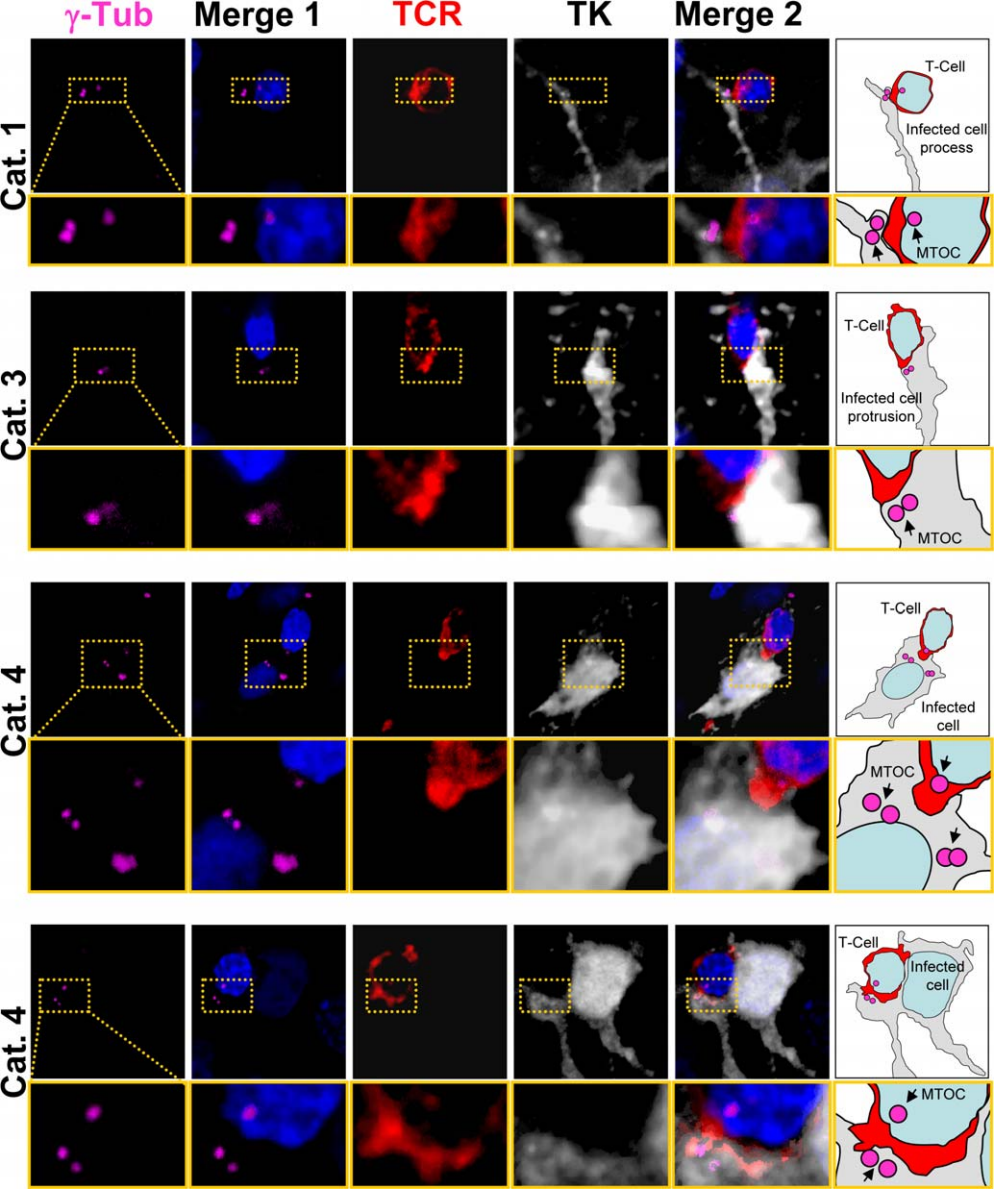
The MTOC of infected brain cells is polarized to the T cell. Representative confocal images of four contacts between astrocytes and T cells. Cells were labeled with markers for MTOC (γ-Tubulin, magenta), viral infection (TK, white), and T cells (TCR, red); and nuclei are stained with DAPI (blue), using immunofluorescent techniques. MERGE 1 is a composite image showing nuclear staining and MTOC. MERGE 2 shows MTOC, virally infected cells, T cells, and nuclei. A magnification of the contact zone is shown below each panel. The right hand column contains drawings of the MERGE 2 images, indicating the spatial relationship between the MTOC (magenta) of the virally infected cells (grey), and the T cells (red). The first row shows a cell of Category 1. The MTOC is localized in a process of the infected cell. The MTOC of the T cell is facing the MTOC of the infected cell. TCR also is polarized towards the infected process. The second row of images represents a cell of Category 3, with MTOC and TCR polarized towards the T cell. The third row shows a cell of Category 4, also with MTOC polarized to the T cell but positioned to one side of the T-astrocyte interface; notice that this astrocyte appears to have multiple MTOC-like structures. Also the MTOC of the T cell is polarized to the MTOC of the infected cell. The bottom row shows another Category 4 cell immunological synaptic junction.

**Figure 5:**
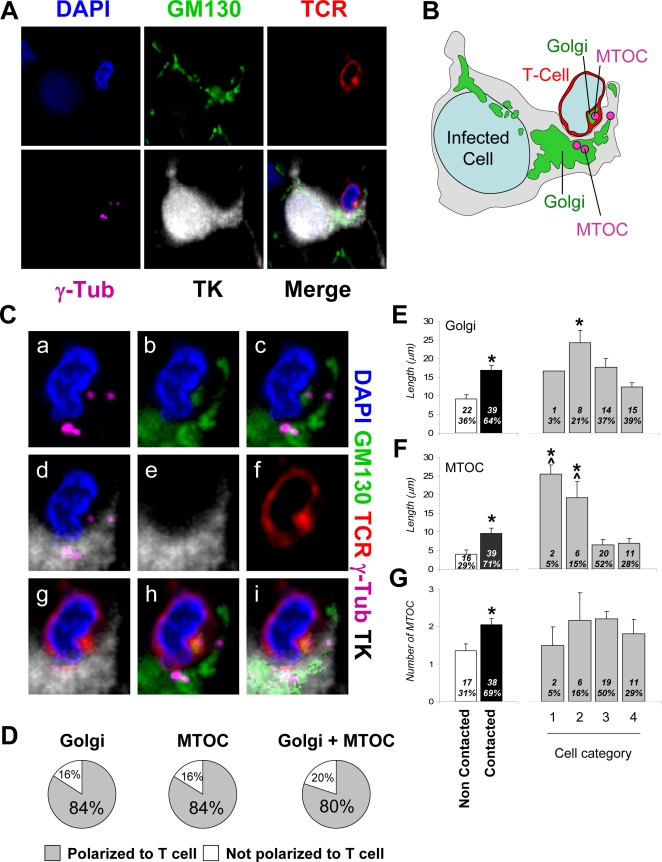
Intracellular distributions of the Golgi apparatus and MTOC are altered in infected brain cells contacted by T cells. A. Confocal images of an infected cell contacted by a T cell, immunofluorescently labeled with markers for MTOC (γ-Tubulin, magenta), Golgi apparatus (GM130, green), viral infection (TK, white), T cells (TCR, red), and with nuclei stained with DAPI (blue). The Golgi apparatus and the MTOC of the virally infected cell are polarized towards the T cell, and the Golgi apparatus and MTOC from the T cell are polarized towards the virally infected cell. TCR displays a pattern typical of the c-SMAC, characteristic of Kupfer-type immunological synapses, and is polarized towards the infected cell. B is a drawing of the cell shown in A. C. Higher magnification images of the T cell contact zone shown in A. MTOC and Golgi apparatus of both cells are facing each other (a, c, d and g–i). The MTOC and the Golgi apparatus are localized in the same area. TCR fluorescence co-localizes with Golgi apparatus and MTOC in the T cell (a–i). D shows the percentage of virally infected brain cells with Golgi and/or MTOC polarized towards the T cell. More than 80% of infected astrocytes forming immunological synapses show evidence of Golgi and/or MTOC polarization. (E) shows results of quantification of length of Golgi apparatus; the left histogram shows that it is significantly longer in contacted than in non-contacted cells (*p<0.05, Student's t test). Among contacted cells, it is significantly longer in Category 2 cells compared to the other categories (*p<0.05, One-way ANOVA). (F) shows the distance of the MTOC from the nucleus of the infected cells, and reveals that this distance is larger in the contacted than in non-contacted cells (*p<0.05, Student's t test), and among contacted cells is larger in categories 1 and 2 than in 3 and 4 (ˆ* p<0.05 vs. Category 3, non-parametric Kruskal-Wallis test followed by Dunn's post-test; ˆ* p<0.05 vs. Category 4, non-parametric Kruskal-Wallis test followed by Dunn's post-test). (G) shows the number of MTOC found in the infected cells and suggests that this is greater in contacted than in non-contacted cells (*p<0.05, Student's t test).

In cells with no CTL contact, the Golgi apparatus was confined to the cell body and located around the nucleus (Figure 3, non-contacted). However, when CTLs were in contact with virally infected astrocytes, the Golgi apparatus in the astrocytes was found to be polarized towards the T cell [Figure 3, Categories 2–4; the MTOC, the organizer of epithelial cell polarity, was similarly polarized (Figure 4)]. When the CTL contact was located at a significant distance from the cell body of a virally infected astrocyte, the Golgi apparatus could be observed along the protrusion, extending towards the CTL (Figure 3, Category 2; and quantification shown in Figure 5E). When the CTL contact was found apposed to the cell body of the virally infected astrocyte, the Golgi apparatus was polarized towards the interface between the virally infected astrocyte and CTL (Figure 3, Category 3). In cases where the contact between the CTL and the cell body of virally infected astrocyte was so close as to exclude cellular cytoplasm from this contact, the Golgi apparatus was found displaced to one site of the contact (Figure 3, Category 4).

#### ii Microtubule Organizing Center

In the absence of CTL contact with virally infected astrocytes, the MTOC, defined as the area of γ–tubulin immunoreactivity, was perinuclear (data not shown). When CTLs were in contact with virally infected astrocytes, there was an increase in the number of MTOC per astrocyte; an average number of 2 MTOCs per astrocyte was detected (Figures 4 and 5G). Specifically, infected astrocyte MTOCs were located to the area of the process in contact with the CTL; at high power the MTOC of both the astrocyte and T cell were found across the immunological synaptic junction (Figures 4, 5). In astrocytes extending a protrusion towards the CTLs, a MTOC could also be found directly opposed to the CTL (Figure 4 Category 1, Figure 5). Also, and similar to the distribution of the Golgi apparatus, if the CTL-astrocyte contact occurred at the astrocyte cell body, the MTOC was opposed, or adjacent, to the contact site (Figure 3 Categories 3 and 4, Figure 5). Indeed, the Golgi apparatus and MTOC were usually found in the same area of the virally infected astrocyte (Figure 5C, D) and were typically facing the Golgi apparatus and MTOC of the CTL across the immunological synaptic junction (Figures 4 and 5).

#### iii Astrocyte plasma membrane proteins

To assess whether CTL contacts caused the reorganization of infected astrocyte plasma membrane proteins, the distribution of various plasma membrane proteins was examined Specifically, we studied the distribution of proteins known to be enriched in the astrocyte membrane, i.e. GLT-1, connexin 43, aquaporin-4, GLAST, and mGluR5; in addition we also studied the distribution of MHC-I because of its importance in immunological synapse formation.

**Figure 6:**
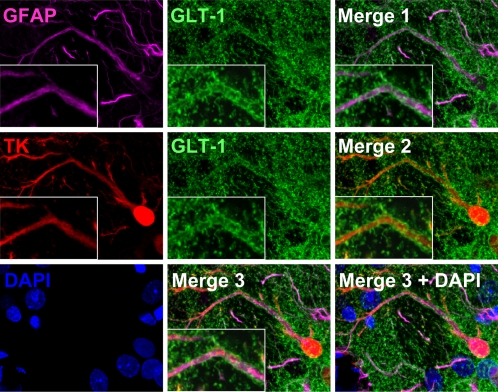
The glutamate transporter GLT-1 is expressed in virally infected astrocytes. Representative confocal images of brain sections stained with markers specific for activated astrocytes (GFAP, magenta), glutamate transporter (GLT-1, green), viral infection (TK, red), and nuclei (DAPI, blue). Merge 1 displays GFAP and GLT-1 immunoreactivity to show expression of GLT-1 in astrocytes; Merge 2 shows TK and GLT-1 immunoreactivity to indicate GLT-1 expression in virally infected cells; and Merge 3 depicts GFAP, GLT-1, and TK immunoreactivity to demonstrate expression of GLT-1 in virally infected astrocytes. White rectangles show a higher magnification of a triple-labeled process. Note that GLT-1 is expressed in astrocytes, and appears localized to the membrane surrounding GFAP filaments.

**Figure 7:**
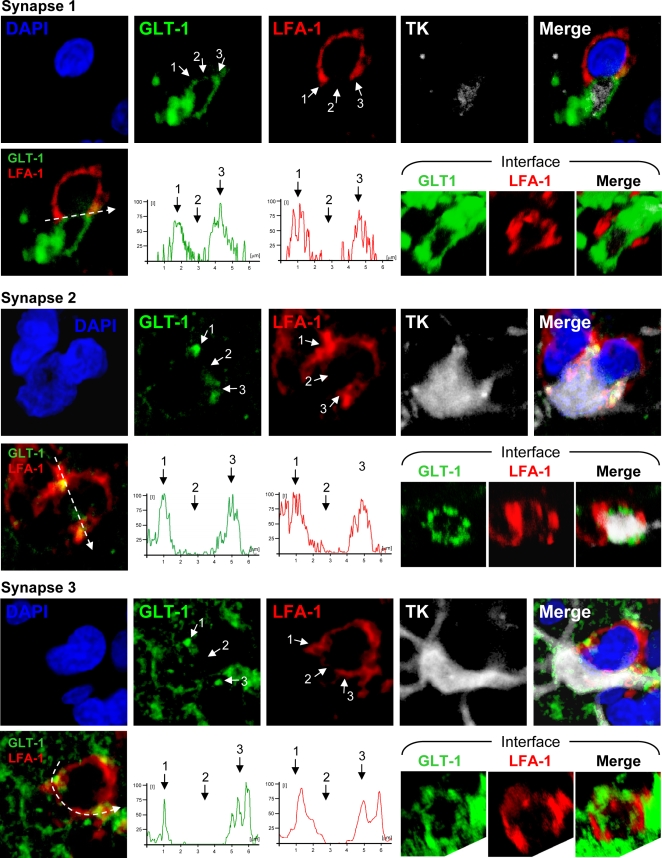
The glutamate transport protein GLT-1 is excluded from the astrocyte membrane area directly opposed to the c-SMAC zone of the Kupfer-type immunological synapse. Confocal images of three Kupfer-type immunological synapses are shown. The top row for each synapse shows images of markers specific for nuclei (DAPI, blue), glutamate transporter (GLT-1, green), T cell adhesion protein (LFA-1, red), viral infection (TK, white), or the superposition of all four color channels (Merge). Bottom rows show fluorescence intensity graphs of GLT-1 and LFA-1 along the path indicated by the white arrow. 3-D reconstructions of the interface of the immunological synapse are shown on the right of the bottom rows with markers specific for GLT-1 and LFA-1. Note the characteristic peripheral distribution of LFA-1 on the T cell (p-SMAC) and a similar peripheral distribution of GLT-1 on the post-synaptic astrocyte. Fluorescence intensity graphs confirm a similar peripheral distribution pattern of LFA-1 and GLT-1. The peripheral area of the interface is indicated by arrows 1 and 3, and the center of the interface by arrow 2 in both confocal images and fluorescence intensity graphs.

GLT-1 is a glutamate transporter, the activity of which is important for astrocytes to maintain normal extracellular medium levels of glutamate [41]. As expected, GLT-1 was expressed by infected astrocytes in the striatum (Figure 6), located diffusely throughout the astrocytic processes. We further examined the distribution of GLT-1 specifically in relationship to immunological synapses (defined by the distribution of LFA-1 to the peripheral-supramolecular activation cluster (p-SMAC), surrounding the central part of the immunological synapses (c-SMAC), [10], [39]). At immunological synapses, the distribution of GLT-1 adopted a particular morphological distribution, i.e. GLT-1 was absent from the area of the astrocyte membrane opposed to the c-SMAC (identified by the absence of LFA-1), but could be found elsewhere on the astrocyte membrane (Figure 7). The semiquantitative analysis of fluorescence intensity indicates that GLT-1 accumulated at the area of astrocyte membrane opposing the p-SMAC and was excluded from the area facing the central portion of immunological synapses (c-SMAC) (Figure 7). Notice the corresponding fluorescence intensity of LFA-1 and GLT-1 in three immunological synapses illustrated.

**Figure 8:**
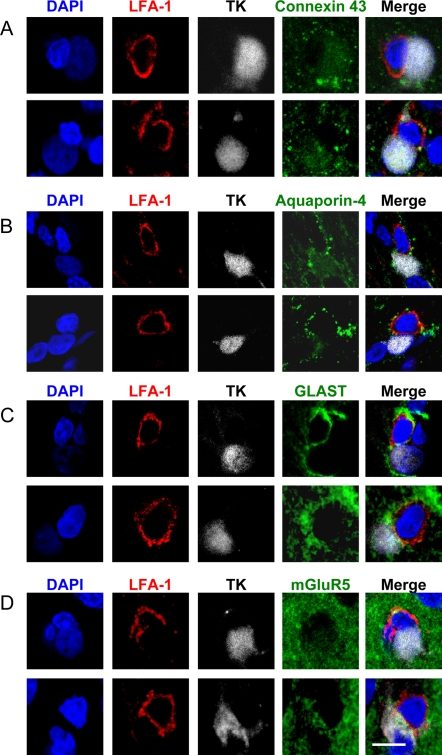
The distribution of other membrane proteins (Connexin 43, Aquaporin-4, GLAST, mGluR5) enriched within the plasma membrane of astrocytes show no obvious pattern of distribution in relation to the T cell immunological synapse. Confocal 0.5 μm optical sections of two immunological synapses are shown stained for each of the membrane proteins. Staining for LFA-1 (red), TK (grey) and DAPI (blue) were combined either with Connexin 43 (green in A), Aquaporin-4 (green in B), GLAST (green in C) or mGluR5 (green in D). The superposition of all four channels is also shown for each synapse (Merge). There was no specific polarization of any of these membrane proteins. Scale bar = 10 μm.

Other plasma membrane proteins examined revealed no particular patterns of distribution in relation to immunological synapses of contacted cells (Figure 8). We interpret this as supporting the hypothesis that the particular distribution of GLT-1 is related to the structure of immunological synapses between T cells and astrocytes. The functional consequences of this phenomenon remain to be determined.

**Figure 9:**
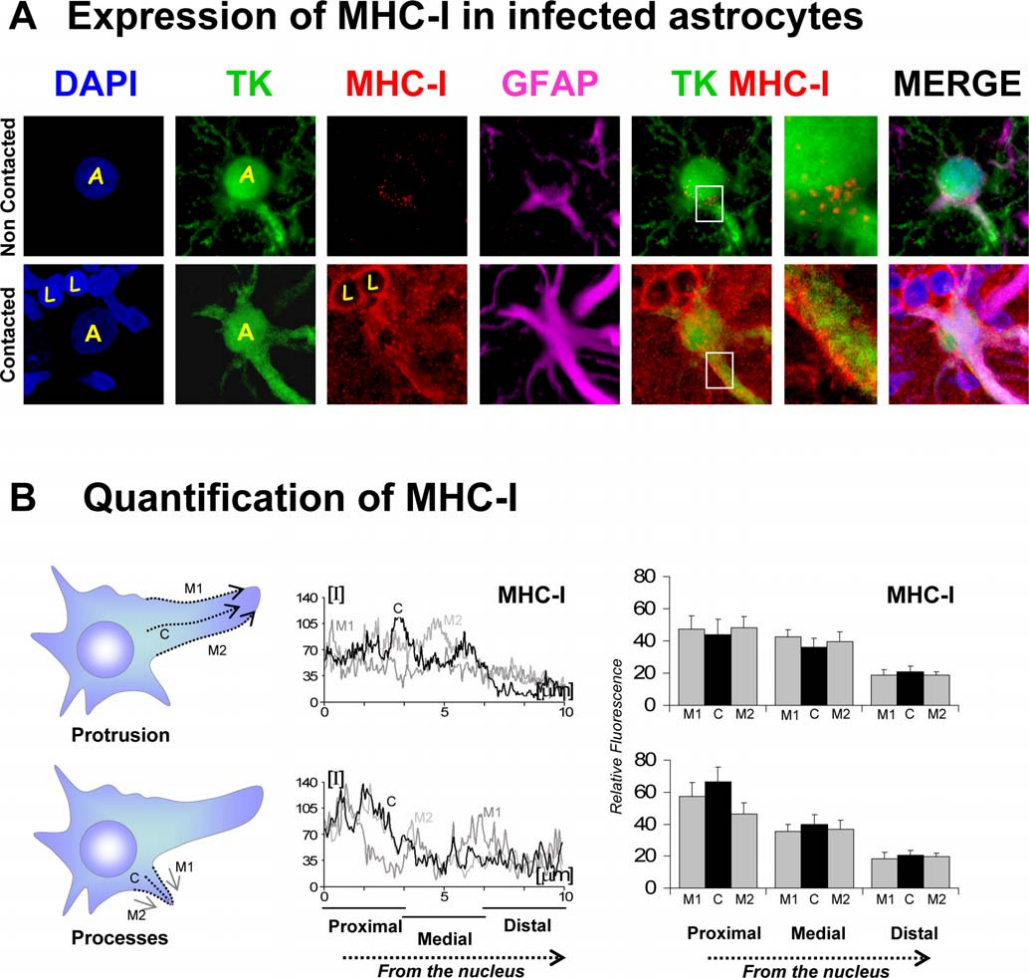
MHC-I expression increases in infected astrocytes during an antiviral immune response, but does not show any particular pattern of distribution in relation to T cells' contacts. (A) shows representative confocal images of non-contacted (top row) and contacted cells (bottom row) stained with DAPI (blue), and immunolabeled with markers of viral infection (TK, green), MHC-I (red), and activated astrocytes (GFAP, magenta). The fourth panel in each row shows an overlapping image of TK and MHC-I (TK MHC-1), of which the fifth panel is a higher magnification of the white boxed area. The last panel is a superposition of all four color channels (MERGE). MHC-I is expressed at high levels in virally infected astrocytes in contact with T cells. Note that presumed leukocytes (identified by the absence of TK, or GFAP immunoreactivity, ‘L’) display intense MHC-I immunofluorescence. Nuclei of virally infected astrocytes are indicated by (yellow) ‘A’. (B) shows the relative quantification of MHC-I fluorescence along protrusions (top panels) or processes (bottom panels) of contacted cells. Illustrations on the left indicate the morphological position of the measurements analyzed (with respect to the nucleus). Fluorescence intensity graphs in the center display relative MHC-I immunofluorescence (measured from the cell body to the cell periphery along the arrows indicated in the left panels). MHC-I intensity on both sides (M1 and M2) and cytoplasm (C) of virally infected cells is shown. Analysis of relative fluorescence on the right shows the average intensity of MHC-I expression in proximal, medial and distal areas, relative to the nucleus. The fluorescence intensity of 30 cells was measured. The expression of MHC-I follows a proximo-distal pattern of intensity.

In virally infected astrocytes, that were yet not-contacted by T cells, MHC-I, a membrane protein central to the interactions between the T cells and the infected astrocytes, was expressed at very low levels. In contacted cells, however, there were high levels of expression of MHC-I (Figure 9A). It was found that the cell body expressed the highest levels of MHC-I. It was also found that the lowest levels of MHC-I were found in the extreme terminals of the processes and that there was a decreasing gradient of MHC-I expression going from the cell body to the terminal process of the astrocyte (Figure 9B); we failed to detect a pattern of distribution of MHC-I which related to the position of T cells contacting the infected astrocytes, and there was no difference between MHC-I expression in processes vs. protrusions. In accordance with an increase in GFAP expression in models of astrocyte injury [7]–[9], [42], expression of GFAP is dramatically upregulated in contacted cells (Figure 9A/GFAP).

### CD8^+^ T cells, but not infected astrocytes display the apoptotic marker activated caspase-3 in the brains of adenovirus-injected animals

**Figure 10:**
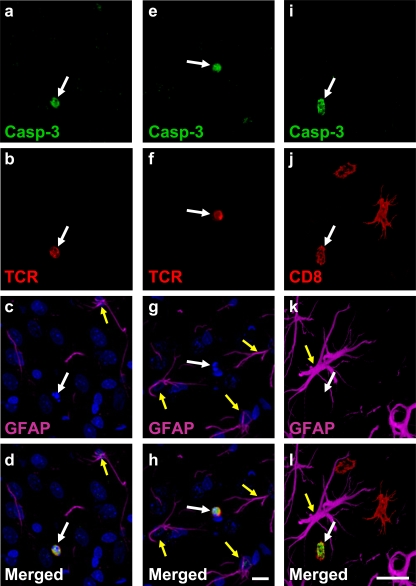
CD8^+^ T cells, but not infected astrocytes display caspase-3 activation following immunization. Figure 10 illustrates the striatum from rats injected intracerebrally with Ad-TK and systemically immunized against adenovirus, and immunolabeled to detect potential apoptosis of infected cells. Tissue sections were labeled with antibodies that recognize activated caspase-3 (green), the T cell markers TCR or CD8 (red), the astrocyte marker GFAP (magenta), and DAPI (blue). Numerous GFAP-immunolabeled astrocytes (yellow arrows) and TCR+ or CD8^+^ T cells (white arrows) were examined; only T cells showed caspase-3 activation (white arrows). Scale bar (a–h; shown in h) = 10 μm; scale bar (i–l; shown in l) = 20 μm.

Immunofluorescent labeling for activated caspase-3, a marker of activation of intracellular apoptosis pathways, revealed a small number of presumably apoptotic cells in the striata of infected animals. Simultaneous labeling with the astrocytes marker GFAP and the T cell markers TCR or CD8 revealed that the cells undergoing apoptosis were exclusively T cells. No caspase-3 activation was observed in GFAP-expressing cells (Figure 10).

### Interactions comparable to those described in the brain in vivo are observed between primary cultured astrocytes and allogeneic T cells in vitro

**Figure 11:**
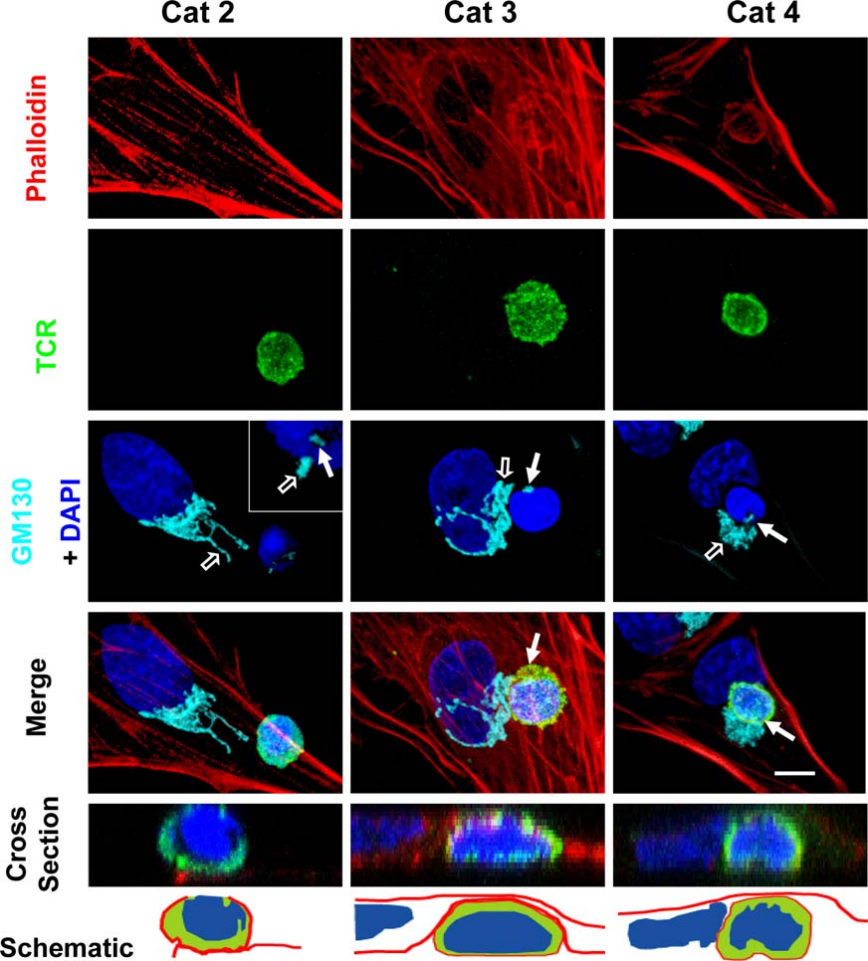
Immunological synapses between allogeneic T cells and Sprague-Dawley astrocytes *in vitro*: in T cells, the Golgi apparatus and T cell receptor polarize towards the target astrocytes, and in target astrocytes, the Golgi apparatus polarizes toward the immunological synapse. This figure shows confocal images of primary cultured astrocytes from neonatal rat cortex derived from outbred Sprague Dawley rats interacting with allogeneic T cells from adult Lewis rats (last column), or outbred Sprague Dawley rats (columns 1 and 2); note that since Sprague Dawley rats are outbred cells from different individuals are allogeneic to each other. One such interaction is shown for each of categories 2, 3 and 4 (as described in Figure 2A for the *in vivo* interactions). Actin is labeled with phalloidin (red), and nuclei with DAPI (blue). TCR and GM130 are immunofluorescently labeled and shown in green and cyan, respectively. The Golgi apparatus of T cells is indicated by a full white arrow; the Golgi apparatus of the target astrocytes is indicated by a white arrow outline. The bottom three panels are vertical cross sections through the T cells, revealing that in the Category 2 interaction, the T cell is superficially apposed to the target, while in the Category 3 and 4 interactions, the T cell is deeply embedded in the target. In the Category 2 interaction, the Golgi body appears to be streaming into the process contacted by the T cell. In the Category 3 interaction, the Golgi is focused in the space between the two nuclei, and in the Category 4 interaction, the T cell is interposed between the nucleus and the Golgi apparatus of the target cell. In the third and fourth panel of each column the Golgi apparatus of the T cell is indicated with a white arrow (this indicates the location of the presumed immunological synapse; for the interaction illustrated for Category 2, the inset illustrates an optical section through the lower part of the T cell actually contacting the astrocyte–due to this anatomical arrangement, the Golgi apparatus appears to be surrounded by the T cell nucleus-). All categories of interaction were detected in the Lewis-Sprague Dawley or Sprague Dawley-Sprague Dawley combinations. In the majority of interactions in which the target Golgi displays the morphology depicted here, the T cell Golgi and the greatest accumulation of TCR immunofluorescence (i.e., the immunological synapse) are co-localized and in contact with the target cell; the TCR polarization can be seen clearest in the fifth panel (cross-section) of the left hand column, and the large accumulation of TCR immunoreactivity towards the intercellular contact seen in the fourth panel (merge) of the middle column. The sixth (bottom) panel of each column is a schematic rendition of the cross section shown in the fifth panel, to illustrate the close spatial relationship between the T cells and allogeneic astrocytes. Scale bar = 6 μm.

Allogeneic T cells, were added to primary cultured astrocytes (from Sprague-Dawley animals); allogeneic T cells were either from inbred Lewis rats, or from a different Sprague-Dawley individual (given that Sprague-Dawley rats are outbred, T cells from two individuals are allogeneic to each other). In this system T cells recognize allogeneic MHC-I, and thus establish immunological synapses with potentially foreign cells. We performed this experiment to determine whether under these conditions allogeneic T cells would establish contacts with astrocytes, in a similar manner to that determined to occur *in vivo,* between brain-infiltrating T cells and virally infected astrocytes. As seen in the brain, the contacts display diverse types of morphological apposition (Figure 11). In this allogeneic interaction between T cells and astrocytes, the Golgi apparatus of astrocytes becomes polarized towards the contacting T cell in a manner and type reminiscent of those interactions described *in vivo* (see Figure 3).

## Discussion

Astrocytes play crucial roles in the maintenance of neuronal structure and function. As such, they respond actively to brain injury caused by trauma, stroke or various neuronal degenerations[5]–[9], [42], [43]. Astrocytes responding to injury *in vivo*, increase in size and process number, increase expression of the astrocyte specific intermediate filament protein GFAP, i.e. they hypertrophy; these astrocytes are described as reactive and/or activated[44]. In all astrocyte responses to injury *in vivo* described so far, astrocytes have been shown to increase in size and process number. We herein describe a novel *in vivo* and *in vitro* injury response of astrocytes reacting to a T cell attack; we demonstrate that under immune attack astroctyes undergo specific morphological changes in response to the formation of Kupfer-type immunological synapses *in vivo* [10], [39]. Virally infected astrocytes in contact with a T cell reduce their total number of processes, form a major protrusion, and re-orient this protrusion, as well as the Golgi apparatus and MTOC towards the contacting T cells. Virally infected astrocytes under immune attack exhibit higher levels of expression of GFAP compared to infected astrocytes from non-immunized animals. While expression levels of activation markers of astrocytes under immune attack are consistent with previously published models of brain injury, we show evidence that the morphological changes by which astrocytes respond to an immune attack are distinct and represent a novel *in vivo* (and *in vitro*) injury response. A similar response was detected in astrocytes in contact with allogeneic T cells, suggesting that the T cells may induce the target astrocytes to polarize towards the contacting T cell. Thus, the capacity of T cells to induce polarization of target cells may be a general phenomenon. This is supported by previous findings showing that during spread of HTLV-1 from an infected to a naïve CD4^+^ T cell, the virus induces polarization of the cytoskeleton of the infected cell, and the infected T cell appears to induce polarization of the target cell towards the infected cell. In this case cytoskeletal repolarization of both CD4^+^ T cells appears to allow the directed intercellular transfer of HTLV-1[45].

Studies on immunological synapses so far have mainly concentrated on the structural and functional reorganizations occurring in T cells. Formation of Kupfer-type immunological synapses is driven by the binding of TCR to cognate antigen on MHC, and the induction of consequent intracellular signaling, phosphorylation, and redistribution of tyrosine kinases such as Lck and ZAP-70 [10], [46]–[50]; this process eventually leads to the polarized secretion of cytokines, such as IFN-γ and effector molecules such as perforin or granzymes [16], [31], [39], [51]–[53]. Much less is known about the cells targeted by CTLs. The consequences of T cells' action on target astrocytes is explored by our work.

We have previously demonstrated that during the clearance of adenovirally infected cells from the brain, CD8^+^, but not CD4^+^ T cells, invade the brain parenchyma where infected astrocytes are located and eliminate approximately 50% of the infected astrocytes (in this paradigm, more than 85% of infected brain cells are astrocytes) [10], [15]. We have also demonstrated that, during such clearance, CD8^+^ T cells form immunological synapses with targeted astrocytes; furthermore, we have also shown that effector molecules of T cells, such as IFNγ and granzyme-B also become polarized at the immunological synapses [15]. Herein we have now analyzed how virally infected astrocytes in the brain *in vivo* respond to the formation of immunological synapses established by antiviral T cells *in vivo*, or how astrocytes respond to the attack by allogeneic T cells *in vitro.*

To explore the interaction between brain-infiltrating T cells and infected astrocytes *in vivo* we used a well-known model in rats in which adenovirally infected astrocytes in the brain are cleared by T cells following systemic immunization against adenovirus. T cells are specifically activated against adenovirus and form Kupfer-type effector immunological synapses with infected astrocytes [10], [14], [39]. In the present work we analyze the morphological changes induced in virally infected astrocytes following the formation of Kupfer-type immunological synapses in the context of immune-mediated clearance.

While T cells' Golgi apparatus and MTOC are relocated towards the intercellular junction in T cells establishing immunological synapses [27], [28], analogous cytoskeletal and organelle reorganization of target cells post-synaptic to CTL Kupfer-type immunological synapses has not been studied. To our knowledge, few studies have examined the effects of Kupfer-type immunological synapses on dendritic cells; these studies demonstrated that dendritic cell reorganization in response to T cell input is Rac-dependent, and that T cells organize the targeting of MHC-II to the dendritic cell plasma membrane [54]. In addition, it has been shown that HTLV-1 appears to use cytoskeleton polarization towards uninfected cells, as a mechanism for targeting uninfected cells; the infected cell polarizes its cytoskeleton towards the uninfected cell, and this is then used by HTLV-1 to move close to, and infect, the target cell [45].

Polarization of astrocytes has previously been described using an *in vitro* lesion model [55]–[59], but has not been described in response to an immune T cell attack. In response to a physical lesion to a confluent monolayer, primary astrocytes develop a protrusion and reorient their MTOC and Golgi towards the lesion [55]–[59]. Such polarization involves the actin cytoskeleton and myosin, and is mediated by a family of Rho GTPases including Cdc42, Rac, and Rho which coordinate the formation of a protrusion, the cellular polarization, and positioning of the astrocyte cytoskeleton, MTOC and Golgi apparatus [55]–[58], [60]–[63]. Cdc42 plays a key role in the polarized positioning of the MTOC, Golgi reorientation, and protrusion formation in astrocytes [56]–[59], [63], [64]. Cdc42 coordinates downstream effectors involving PKCζ to drive MTOC positioning, as well as a second cascade involving Rac which guides protrusion formation [57], [59], [64]. Whether these cascades are involved in the morphological rearrangements of infected astrocytes forming immunological synapses with T cells *in vivo*, or astrocytes being contacted by allogeneic T cells *in vtro*, is currently being evaluated.

The actual function of astrocyte morphological polarization in response to CTL attack and formation of an immunological synapse remains to be determined. Polarization of T-cells involved in immunological synapses is thought to underlie their effector functions [17], [25], [31], [65], [66]. It has been previously demonstrated *in vitro* that MTOC is polarized in T cells towards target cells; this polarization drives the secretion of granules at the interface of the immunological synapse of cytotoxic T lymphocytes [28], [29], [34], [36], [67], and intercellular transfer of HTLV-1 between CD4^+^ T cells [45]. Kupfer-type immunological synapses direct the polarization of IFN-γ and granzyme B towards the synapse in order to be secreted towards target cells *in vivo* [39].

It is tempting to speculate that T-cells bind preferentially to the distal portion of astrocytes. This may lead to changes in astrocyte morphology (and presumably function) such as described herein. How the morphological changes ultimately influence the capacity of T cells to kill infected astrocytes, or the capacity of astrocytes to withstand T cell cytotoxic attack, remains to be determined. It is interesting to note that in our rat model T cells only eliminate approximately 50% of infected astrocytes. Further, astrocyte polarization is an active phenomenon, and, in epithelial cells, polarization usually indicates the direction in which polarized cells will move towards. As neuroepithelial-derived astrocytes detect the T cell attack, it is possible that they respond by moving towards the T cells to surround them, and thus block them from attacking further cells. A relatively poorly studied phenomenon, known as emperipolesis [68]–[70], describes the capacity of brain glial cells to ‘take up’ lymphocytes. In fact, a re-examination of T cell-astrocyte interactions we described previously *in vivo*, and was published in Figures 7 and 8 of our previous publication [10], [39], are compatible with this notion. Thus, it would be possible, that emperipolesis is another mechanism by which brain astrocytes down-regulate immune attack of the brain. The molecular and functional mechanisms of astrocyte emperipolesis of attacking T cells are currently under examination by us. Interestingly, target lymphocytes of CD4^+^ cells infected with HTLV-1 also polarized their cytoskeleton towards the infected cell. Thus, it is possible that our data are part of a general phenomenon by which T cells (whether anti-viral, anti-transplant, autoimmune or anti-tumor) induce the polarization of target cells. We are currently working to discern the molecular basis of such responses in astrocytes.

In summary, our results suggest that the formation of immunological synapses between T cells and astrocytes induces dynamic changes in target astrocytes, both *in vivo* and *in vitro*. Further, we demonstrate that target cells, in response to T cell attack and formation of immunological synapses, rearrange their morphological phenotype, redistribute their MTOC, reduce the number of processes and form a protrusion which contains extended Golgi stacks, and apparently, a duplicated MTOC. These results suggest that the formation of immunological synapses affects target cell structure. Astrocytes targeted by the immune system display classical signs of injury (GFAP upregulation), however, they respond to T cell attack in a novel fashion by extending a major protrusion towards the immunological synapse formed by the effector T cells, and withdrawing most of their finer processes. This response to injury differs from the hypertrophic response mechanism to other forms of brain injury, and thus we believe it constitutes a novel response mechanism to injury. We believe this may have an important role in the immune mediated clearance of virally infected cells from the brain in HIV/AIDS, HTLV-1, HSV-1, West Nile virus, infections, in the immune responses to brain cells transduced with gene therapy viral vectors, or during autoimmune, anti-transplant, or anti-tumor immune responses in the brain. The molecular mechanisms underlying astrocyte responses to T cell attack *in vivo* and *in vitro*, and the functional consequences thereof, are currently under investigation.
